# NSAID exposure delays time-to-pregnancy in patients with spondyloarthritis: an analysis of the GR2 prospective cohort

**DOI:** 10.1136/rmdopen-2024-004745

**Published:** 2024-11-29

**Authors:** Sabrina Hamroun, Marion Couderc, René-Marc Flipo, Jérémie Sellam, Christophe Richez, Emanuelle Dernis, Aline Frazier, Laure Gossec, Elisabeth Gervais, Hubert Marotte, Laetitia Dunogeant, Cédric Lukas, Alban Deroux, Gaëlle Guettrot-Imbert, Véronique Le Guern, Nathalie Costedoat-Chalumeau, Anna Molto, On behalf of the GR2 study group, Béatrice Banneville

**Affiliations:** 1 Rheumatology Department, HP center, Paris, France; 2 Université Paris Cité, Paris, France; 3 Rheumatology, University Hospital Centre, Clermont-Ferrand, France; 4 Inserm/Imost UMR1240, Clermont Auvergne University, Clermont-Ferrand, France; 5 Université Lille 2 Droit et Santé BU Santé Learning Centre, Lille, France; 6 Rheumatology, INSERM UMRS_938, Sorbonnes Université UPMC Univ Paris 06, St-Antoine Hospital, DHU i2B, Paris, France; 7 Service de Rhumatologie, Hôpital Pellegrin, CHU de Bordeaux, Bordeaux, France; 8 UMR CNRS 5164, Université de Bordeaux Collège Sciences de la Santé, Bordeaux, France; 9 Rheumatology, Le mans hospital, Le Mans, France; 10 Service de rhumatologie, AP-HP, Hôpital Laribosière, Paris, France; 11 INSERM, Institut Pierre Louis d'Epidémiologie et de Santé Publique, Sorbonne Universite, Paris, France; 12 Rheumatology Department, APHP, Hopital Universitaire Pitie Salpetriere, Paris, France; 13 CHRU de Poitiers, Poitiers, France; 14 Rhumatologie, CHU Saint-Etienne, Saint-Etienne, France; 15 SAINBIOSE, INSERM U1059, University of Lyon, Saint-Etienne, France; 16 Rheumatology and Internal Medicine Department, Pays d’Aix Hospital, Aix-en-Provence, France; 17 Rheumatology, University Hospital Lapeyronie, Montpellier, France; 18 EA2415, Montpellier University, Montpellier, France; 19 Internal Medicine Department, Grenoble, Grenoble, France; 20 Department of Internal Medicine, Université de Paris, Paris, France; 21 Department of Internal Medicine, APHP, Paris, France; 22 Medecine Interne Cochin Hospital, Paris, France; 23 Rheumatology, Hospital Cochin, Paris, France; 24 INSERM U1153, CRESS - Université Paris-Cité, Paris, France

**Keywords:** Spondyloarthritis, Epidemiology, Anti-Inflammatory Agents, Non-Steroidal, Axial Spondyloarthritis

## Abstract

**Background:**

The impact of disease activity and treatment on fertility outcomes in patients with spondyloarthritis (SpA) has been little explored. This study aimed to describe median time to pregnancy (TTP) in women with SpA and the factors influencing TTP in this population.

**Methods:**

This prospective observational multicentre (63 centres) French cohort (GR2 study—NCT02450396) included consecutive women with a diagnosis of SpA (according to their rheumatologist) who wanted to become pregnant between 2015 and 2021. TTP (in months) was the main outcome criterion, prospectively calculated from the date of study inclusion to the date of conception. Data on demographics, disease characteristics, disease activity, severity and treatment were prospectively collected at inclusion and each year thereafter until pregnancy occurred. TTP and its associated factors were estimated by survival analysis (Shared Frailty Cox models), with a random centre effect and multiple imputation to address missing data.

**Results:**

We analysed 88 women included before conception. Among them, 56 (63.6%) became pregnant during follow-up. Median TTP was 16.1 (95% CI (12.2 to 25.3)) months. Mean preconceptional Bath Ankylosing Spondylitis Disease Activity Index at inclusion was 2.9 (±SD 2.1). Patients were treated with TNF inhibitors, non-steroidal anti-inflammatory drugs (NSAIDs), conventional synthetic disease-modifying antirheumatic drugs and glucocorticoids in 61 (69.3%), 23 (26.1%), 12 (13.6%) and 8 (9.1%) cases, respectively. The multivariate model found a significant association between TTP and age (HR) (per year) 1.22 95% CI (1.08 to 1.40); p<0.001) and the use of NSAIDs during preconception (HR 3.01 95% CI (2.15 to 3.85); p=0.01).

**Conclusion:**

Age and NSAID use during preconception were significantly associated with a longer TTP, after adjustment for other confounding factors. These findings warrant caution in the use of NSAIDs in SpA patients trying to conceive.

**WHAT IS ALREADY KNOWN ON THIS TOPIC**

- Prior to this study, there were limited data on the impact of disease activity and treatments on fertility outcomes in patients with spondyloarthritis (SpA). While the negative effects of non-steroidal anti-inflammatory drugs (NSAIDs) on fertility were known in other chronic rheumatic inflammatory diseases, such as rheumatoid arthritis, little was understood about their impact on fertility in SpA patients. Additionally, previous studies on fertility in SpA were retrospective, had a high risk of bias and often did not focus on the specific fertility challenges faced by women with SpA. Therefore, a prospective, detailed evaluation of factors influencing time to pregnancy (TTP) was necessary.

**WHAT THIS STUDY ADDS**

- This study provides the first prospective evaluation of TTP in women with SpA. The findings highlight that subfertility is common, with 45% of the women classified as subfertile and a median TTP of 16.1 months. The study also identifies NSAID use during preconception as a significant factor associated with delayed TTP, alongside maternal age. These results emphasise that NSAIDs can significantly prolong TTP, confirming concerns about their potential negative effects on fertility in women with SpA.

**HOW THIS STUDY MIGHT AFFECT RESEARCH, PRACTICE OR POLICY**

- The implications of this study are significant for clinical practice, particularly in advising women with SpA who wish to conceive. The findings suggest the need for caution when prescribing NSAIDs during the preconception period and underline the importance of preconception counselling in this population. Future research should explore the mechanisms behind NSAIDs’ impact on fertility and assess alternative treatment strategies to support conception in women with SpA.

## Introduction

 Spondyloarthritis (SpA) (both axial and peripheral) is among the most frequent chronic rheumatic inflammatory diseases (CRID), particularly in people of childbearing age.^1 2^ This disease causes pain, stiffness and major disability in patients in their reproductive years. Left untreated, SpA may lead to severe loss of function in these young patients, with a major impact on their quality of life. SpA has been classically seen as a predominantly male disease, but recent data have indicated little to no differences in the sex ratio prevalence of the disease.^3 4^ Therefore, the issue of desire for pregnancy and treatment management in this situation is an increasingly common question in clinical practice.^5^

Impaired fertility and poorer ovarian function have been reported in patients with other CRID, such as rheumatoid arthritis (RA),^6^,^10^ with longer time to pregnancy (TTP) associated with disease activity but also with preconceptional exposure to glucocorticoids and non-steroidal anti-inflammatory drugs (NSAIDs).^11^ Little is known about the impact of disease activity and treatment strategies on fertility and pregnancy outcomes in SpA.^12^

A systematic literature review^13^ reported only four studies in women with SpA with information on fertility, and all had a high risk of bias^13^ that precluded drawing any conclusions. All were based on retrospective designs, with a risk of recall bias. This was highlighted, for example, in one of them that showed a significant discrepancy between the self-reported data and the medical records.^14^

Other factors are suspected to have a potentially negative influence on fertility in women with SpA. One of these such is the chronic use of NSAIDs, particularly selective COX-2 inhibitors, which can lead to anovulatory cycles due to the luteinised unruptured follicle syndrome.^15^,^17^ However, the potential negative impact of NSAIDs on fertility outcomes has not been evaluated in women with SpA, although it seems particularly relevant as NSAIDs remain the cornerstone treatment in this disease.^18^,^20^

More recently, the Norwegian RevNatus registry showed that TTP exceeded 12 months (the threshold for defining ‘infertility’) in 21% of 274 women with SpA followed up between 2006 and 2018. In this cohort, longer TTP was associated with older age, nulliparity and longer disease duration.^21^

These points led us to conduct this study, which aims to describe TTP in a cohort of women with SpA and to evaluate the association of TTP with disease activity and preconceptional treatments.

## Methods

### The GR2 study

We report data from the GR2 (NCT02450396) study, a French multicentre prospective observational study of women desiring pregnancy and a rare and/or rheumatological diseases, including SpA (both axial and peripheral phenotypes). This study has been underway since October 2014 in 63 centres and is described elsewhere.^22^,^24^ Briefly, women were included by their clinicians (internists or rheumatologists), and treatment decisions were left to these physicians’ discretion. The GR2 study is part of the European network of pregnancy registers in Rheumatology supported by Foundation for Research in Rheumatology^25^ and follows European Alliance of Associations for Rheumatology recommendations regarding core data sets for these registers.^26^

Investigators provided all patients with written information and obtained oral consent from them, in accordance with French regulations for observational non-interventional studies.

### Populations and analysis

Women with SpA (both axial and peripheral phenotypes, diagnosed by their rheumatologist) were included between December 2015 (although inclusion in the overall cohort began in October 2014, inclusion for women with RA and SpA began in December 2015) and June 2021 (this date was chosen to have at least 1 year of follow-up after the inclusion of women desiring pregnancy). Patients could be included in either the ‘preconception’ module or the ‘pregnancy’ module of the study. In the ‘preconception’ module, consecutive women with SpA and desiring pregnancy (who had recently ceased contraception or planned to do so in the near future) were included in the ‘preconception module’ and were followed up yearly until pregnancy occurred or the patient no longer desired to be pregnant or after 5 years of trying to conceive, whichever arrived first. Accordingly, one desired pregnancy corresponds to one inclusion. If a patient was included twice or more for more than one pregnancy, only the first inclusion was analysed. If during follow-up in the ‘preconception’ module a pregnancy was confirmed, patients transferred into the ‘pregnancy’ module, that is, they were followed up at least once per pregnancy trimester and at 12 months post partum. Women with SpA not included in the preconception module who had a confirmed pregnancy at less than 14 weeks of gestation could be immediately included in the ‘pregnancy’ module.

Only patients included in the preconception period (ie, via the ‘preconception’ module) were included in the main analysis. TTP was also calculated in the population of patients included via both modules (‘preconception’ and ‘pregnancy’), both as a sensitivity analysis and to explore if entering the cohort by the ‘preconception’ module created a potential selection bias. That is, we examined the hypothesis that patients who became pregnant immediately after stopping contraception might not have had the time to enter the ‘preconception’ module and were included directly in the ‘pregnancy’ module), as TTP was also collected at the inclusion visit in the latter module, although retrospectively.

### Data collection

At the first visit of the ‘preconception’ module, we collected the following data: demographics, SpA characteristics (including the items enabling calculation of the ASAS (Axial Spondyloarthritis Assessment international Society) criteria^27^), SpA history, current SpA treatment (symptomatic, ie, analgesics, NSAIDs, glucocorticoids and steroidal injections; conventional synthetic disease-modifying antirheumatic drugs (csDMARDs): methotrexate, leflunomide, sulfasalazine, azathioprine and hydroxychloroquine and biological disease-modifying drugs ((b)DMARDs), eg, TNF inhibitors), obstetrical history (any previous pregnancies and their outcomes: early miscarriage, stillbirth or live-birth), treatments received and events during pregnancy (infections/disease flares/others), comorbidities (thrombo-embolic disease, hypertension, diabetes, dyslipidaemia) and status for hepatitis B and C viruses and HIV. At each visit (including the first one), disease activity and its severity, and ongoing treatments were collected at the first visit and every subsequent visit.

### Definitions of outcomes and disease activity

The primary outcome was TTP in months, defined as the time between the cessation of contraception and confirmed pregnancy. The secondary outcome was the number of subfertile patients (ie, patients with a TTP>12 months^28^ or non-achievement of pregnancy). Disease activity was collected by the Bath Ankylosing Spondylitis Disease Activity Index (BASDAI).^29^ C reactive protein (CRP) was collected at each preconceptional visit. Although ASDAS^30^ is the preferred disease activity outcome, we chose to consider BASDAI and CRP separately so that we could assess the potential effect on TTP of both ASDAS components—systemic inflammation with CRP and disease activity burden with BASDAI.

The clinical research form included fillable fields for specifying the start date (either the exact start date if within the past 12 months or the mention of treatment for more than 12 months), the end date (only if it was stopped during the follow-up) and the dose and frequency of intake. Without a treatment start or end date, we considered it was taken before or after the follow-up period, respectively.

### Statistical analyses

First, we described the analysis populations (main and sensitivity analyses) in terms of demographics, disease characteristics, disease activity, severity and treatment history. The different treatment exposures during preconceptional follow-up were described and at least one dose during this period constituted exposure. Continuous variables normally and not normally distributed were expressed, respectively, by their means with SDs and medians with IQRs.

Second, TTP for the main analysis was estimated with survival models (Kaplan-Meier). Moreover, to determine the factors associated with a pregnancy occurrence (ie, the event), we used shared frailty Cox models for the multivariate analysis, including a random centre effect^31^ to consider potential heterogeneity of practices among centres. The explanatory variables were selected based on the univariate analyses and current knowledge of factors potentially associated with fertility disorders. The threshold of p<0.2 in univariate analyses was used determine the introduction of variable in the multivariate model. Several variables were forced into the model because of the strong evidence of their negative impact on fertility: current smoking, disease activity, nulligravidity and body mass index (BMI). Exposure to csDMARDs and TNF inhibitors was also forced into the model to examine the effect of therapeutic classes significantly associated with a longer TTP, independently of exposure to other classes. Results are presented as HRs with their 95% CIs. For the sensitivity analysis, TTP was estimated by combining the prospective observations of the TTP (months) in the ‘preconception’ module and the TTP (months) recalled by the patients, included directly in the ‘pregnancy’ module.

No analysis of associated factors was performed in the sensitivity analysis population, as prospective records of disease activity were not available for them.

Missing data were addressed by using multiple imputation by chained equations (MICE), which involves generating multiple imputed datasets to take into account the uncertainty associated with missing values. The imputation process was performed with 100 iterations to ensure robust and reliable estimates.

All analyses were conducted with R. Studio R V.4.0.3 (2020-10-10) and used the following packages: prettyR, tidyverse, lubridate, dplyr, survival, coxme, survminer and mice.

## Results

### Description of the population

#### Main analysis population

**Figure 1:**
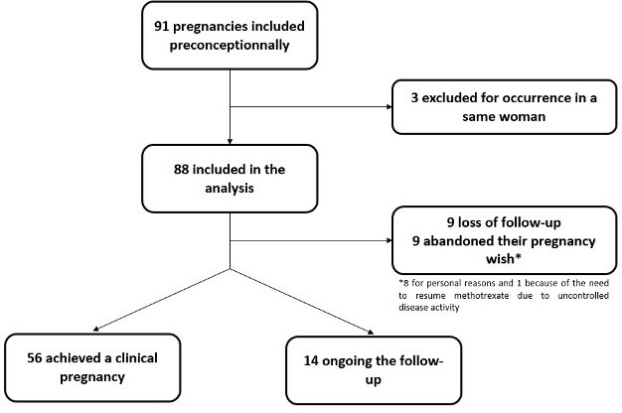
Flow chart of SpA patients included in the preconception period. SpA, spondyloarthritis.

From December 2015 to June 2021, 207 women with SpA were enrolled. Among them, 91 (44.0%) were included in the ‘preconception’ module and had a prospective follow-up during the preconception period; 88 of them were finally retained in the fertility analysis (figure 1). Three patients were included twice in the cohort for two different desired pregnancies; only the first inclusion was analysed (the first pregnancy was considered the event of interest, while the follow-up was censored according to the methodology of survival analyses).

In this 88-patient main analysis population, the mean age was 31.6 (±4.1) years and the mean BMI 23.8 (±4.9) kg/m^2^. 44 (50%) were nulligravid and 17 (19.3%) smokers in the preconception period (table 1). SpA was axial in 36 (40.9%) cases, with a median duration of 5.0 (2.0–9.0) years at inclusion. The extra-articular manifestations included 13 (14.8%) patients with uveitis, 18 (20.5%) with psoriasis and 7 (8.0%) with inflammatory bowel disease. Severity signs were found for a history of coxitis for 3 (3.4%), total hip replacement for 1 (1.1%) and syndesmophytes in 2 (2.3%) cases. 61 women (69.3%) had received at least one TNF inhibitor for SpA, and 10 (11.4%) had at least 3 different TNFis for SpA (table 1).

**Table 1:** Baseline characteristics of SpA patients included during the preconceptional period

	Population (N=88)	Population of the sensitivity analysis (N=143)
Basic characteristics		
Age, mean (SD) years	31.6 (4.07)	31.8 (4.23)
Current smoking (n=81)	17 (19.3%)	20 (14.0%)
Current alcohol consumption (n=77)	3 (3.4%)	3 (2.1%)
BMI, mean (SD) kg/m^2^	23.8 (4.85)	22.7 (4.68)
No previous pregnancy (n=88)	44 (50%)	62 (43.3%)
Disease-related characteristics		
Disease duration, median (IQR)	5.0 (2.0–9.0)	5.0 (2.0–9.0)
History of glucocorticoid use (n=88)	32 (36.4%)	51 (35.7%)
History of csDMARD use (n=88)	37 (42.0%)	60 (42.0%)
Salazopyrine	16 (18.2%)	28 (19.6%)
Methotrexate	31 (35.2%)	43 (30.1%)
History of NSAID use (n=88)	70 (79.5%)	106 (74.1%)
History of TNFi use (n=88)	61 (69.3%)	105 (73.4%)
Only 1 TNFi	36 (40.9%)	60 (42%)
2 TNFi	15 (17.0%)	28 (19.6%)
At least 3 TNFi	10 (11.4%)	14 (9.8%)
SpA phenotype (n=84)		
Axial	36 (40.9%)	63 (44.1%)
Peripheral	21 (23.9%)	34 (23.8%)
Both	27 (30.7%)	38 (26.6%)
History of coxitis	3 (3.4%)	3 (2.1%)
History of total hip replacement	1 (1.1%)	1 (0.7%)
Syndesmophytes	2 (2.3%)	2 (1.4%)
Uveitis	13 (14.8%)	20 (14.0%)
Psoriasis	18 (20.5%)	24 (16.8%)
Inflammatory bowel disease	7 (8.0%)	10 (7.0%)
Comorbidities		
Hypertension	2 (2.3%)	3 (2.1%)
Diabetes	0 (0%)	1 (0.7%)
Polycystic ovary syndrome	1 (1.1%)	2 (1.4%)
Endometriosis	7 (8.0%)	9 (6.3%)

BMIbody mass indexcsDMARDsconventional synthetic disease-modifying antirheumatic drugsNSAIDsnon-steroidal anti-inflammatory drugsSpAspondyloarthritisTNFitumour necrosis factor inhibitor

#### Sensitivity analysis population

Among the 116 SpA patients who were included directly in the ‘pregnancy’ module, information on TTP was available for only 55 patients. The sensitivity analysis population thus included 143 patients. That is, pregnancy was not planned for 19/116 and thus TTP could not be collected for them; overall, 42/116 patients had missing TTP data. The characteristics of the populations in the main and sensitivity analyses were relatively similar (table 1).

### Time to pregnancy

#### Main analysis

Of the 88 women included preconceptionally, 56 (63.6%) achieved a clinical pregnancy and 40 (45.4%) did not and were classified as subfertile. Among these 56, the median TTP was 16.1 months (95% CI (12.2 to 25.3)). On the whole, disease activity was controlled at the inclusion visit with a mean BASDAI of 2.9 (± 2.1). Patients were mainly treated with TNF inhibitors during the preconception period (69.3%), while 23 (26.1%) received NSAIDs. Only 12 (13.6%) were on csDMARDS, and 8 (9.1%) on glucocorticoids (table 2). A description of the NSAIDs used by patients at inclusion is provided in online supplemental table 1.

**Table 2:** Treatment use and disease activity during the preconception period

	Patients (N=88)
Glucocorticoids, n (%)	8 (9.1)
csDMARDs, n (%)	12 (13.6)
TNFi, n (%)	61 (69.3)
NSAIDs, n (%)	23 (26.1)
BASDAI score, mean±SD (n=74)	2.88±2.09
CRP, median (IQR) mg/L (n=53)	2.63 (0.75–7.40)

CRP, C reactive proteincsDMARDs, conventional synthetic disease-modifying antirheumatic drugs; NSAIDs, non-steroidal anti-inflammatory drugs; score, Bath Ankylosing Spondylitis Disease Activity Index score; TNFi, tumour necrosis factor inhibitor

Univariate analyses found p<0.2 for the associations of TTP with preconceptional NSAID exposure, disease duration, age and SpA phenotype. These variables were included in the multivariate model, to which we added csDMARDs and TNF inhibitor exposure during the preconception period, nulligravidity, BMI, disease activity and current smoking, as explained above.

**Figure 2:**
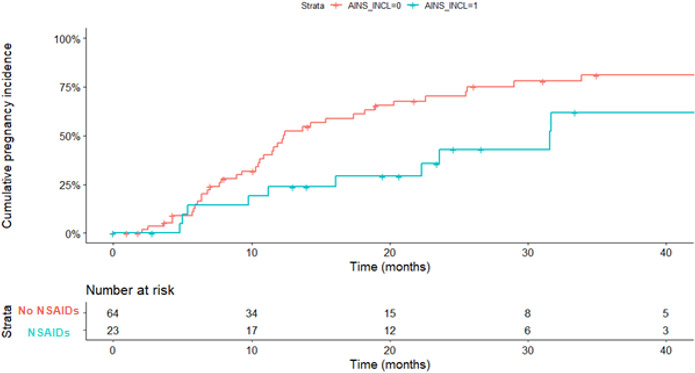
Cumulative pregnancy incidence in women with SpA with and without NSAID use in the preconceptional period. NSAIDs, non-steroidal anti-inflammatory drugs; SpA, spondyloarthritis.

The multivariate shared frailty model (adjusted for age, BMI, nulligravidity, BASDAI at inclusion, disease duration, smoking, SpA phenotype—axial, peripheral or both—and exposure to NSAIDs, csDMARDs and biological treatment in the preconception period) found that TTP was significantly and independently associated with age (HR (per year) 1.22, 95% CI (1.08 to 1.40); p<0.001) and preconceptional NSAID use (HR 3.01, 95% CI (2.15 to 3.85); p=0.01) (table 3). Median TTP was 31.6 months (95% CI (22.3 to 40.4)) in women who were exposed to NSAIDs during the preconceptional period, vs 12.3 months (95% CI (10.9 to 20.3)) in women who were not (p=0.01) (figure 2).

**Table 3:** Cox regression analysis for time to conception in women with SpA included in the preconception period

	Univariate analyses		Multivariate analyses	
	Crude HR 95% CI	P value	Adjusted HR 95% CI	P value
Age	**1.10(1.02to1.19**)	**0.020**	**1.14(1.03to1.26**)	**0.019**
Nulligravidity	1.32 (0.78 to 2.27)	0.320	1.58 (0.86 to 2.91)	0.146
BMI	1.01 (0.95 to 1.06)	0.837	0.99 (0.94 to 1.05)	0.827
Current smoking	1.10 (0.53 to 2.27)	0.803	0.93 (0.44 to 1.96)	0.843
Disease duration	0.96 (0.91 to 1.01)	0.090	0.95 (0.90 to 1.01)	0.112
BASDAI score	1.09 (0.96 to 1.24)	0.194	1.06 (0.91 to 1.23)	0.456
SpA type				
Axial=ref	1		1	
Peripheral	0.93 (0.45 to 1.91)	0.835	1.13 (0.51 to 2.55)	0.761
Both	**0.49(0.27to0.92**)	**0.030**	0.49 (0.24 to 0.99)	0.055
NSAIDs	**2.30(1.18to4.47**)	**0.017**	**2.71(1.24to5.91**)	**0.010**
csDMARDs	1.49 (0.58 to 3.78)	0.410	1.33 (0.48 to 3.67)	0.589
TNFi	0.85 (0.45 to 1.60)	0.618	0.95 (0.64 to 1.78)	0.104

Statistically significant results are presented in bold.

BASDAI scoreBath Ankylosing Spondylitis Disease Activity Index scoreBMI, body mass index; csDMARDs, conventional synthetic disease-modifying antirheumatic drugs; NSAIDsnon-steroidal anti-inflammatory drugsSpA, spondyloarthritis; TNFi, tumour necrosis factor inhibitor

#### Sensitivity analysis

The sensitivity analysis for TTP reassessment in the overall population included patients in the ‘preconceptional’ and those included only after pregnancy onset—for whom TTP was collected retrospectively. Median TTP was 9.1 months (95% CI (6.4 to 11.6)), and the subfertility rate was 33.6%.

## Discussion

Our study is the first to evaluate TTP prospectively in women with SpA, including all of its types. The aim of this study was to describe TTP prospectively in patients with SpA who desired to become pregnant. In the main analysis population (ie, patients with a prospective observation of TTP), the median TTP was 16.1 months and the subfertility rate 45.4%. Factors associated with a longer TTP were age and preconceptional NSAID use.

These results should be interpreted in the light of general population data: a study from 1999 showed a prevalence of subfertility of 16% in a general Western European population (17.2% in a population of 523 French women) and a median TTP of 3 months.^32^ A later study based on population surveys from 25 countries found a mean prevalence of subfertility of 9%.^33^ In a more recent study among a French general population of women of childbearing age who were trying to conceive, 24% did not achieve a pregnancy after 12 months.^34^ Thus, subfertility appears more common in women with SpA, even though we were unable to make a direct comparison with a control group.

We chose to analyse TTP for the assessment of fertility. This method is considered superior to fertility analysis with anti-Müllerian hormone (AMH), due to the low correlation between AMH and the occurrence of pregnancy in healthy populations.^35 36^ AMH has proven its usefulness in cases of infertility to predict response to ovarian stimulation and its use is standard for dose-adjustment of treatment against infertility.^37^,^39^ Studies in healthy populations, however, have not found that its utility extends to them.^40 41^ Nonetheless, these elements must be clarified specifically in women with SpA, as a recent prospective cohort found lower AMH measurements and follicular counts in patients with radiographic axial SpA compared with healthy controls.^42^ Some authors also suggest that TTP use for fertility assessment must always be prospective to avoid recall bias.^43^,^45^

Only one recent study has specifically investigated TTP in women with SpA.^21^ It included 274 women with axSpA and found a median TTP of 2 months, with 21.2% of the women subfertile. Ursin *et al* also found that age, nulliparity and disease duration were associated with a longer TTP. The differences in our results might be explained by differences in our respective populations. Women in our cohort were more likely to be smokers than those in theirs (19.3% vs 6.4%). They also excluded women with a known cause of infertility.

Moreover, our cohort’s recruitment took place mainly in tertiary centres, whereas patients from Ursin’s cohort received follow-up care in all kinds of centres. This might well explain why a history of TNF inhibitor treatment was observed in nearly 70% of our cases, and more than 10% of patients had already received at least three different TNFi at inclusion. By comparison, the proportion of women on TNF inhibitors in the study by Ursin *et al* was 49%, a figure suggesting that the women with SpA in our study had more severe cases.

Our study highlights the negative impact of NSAIDs on the fertility of women with SpA, with a median TTP threefold greater in women exposed to NSAIDs preconceptionally. Their impact on ovulation, implantation^46^ and risk of miscarriage is well documented in the literature,^47 48^ although less well for CRID. They could play a role in subfertility by inhibiting COX-1 and COX-2, which are necessary for follicle wall rupture and ovum release,^49 50^ which may lead to luteinised unruptured follicle syndrome. Furthermore, animal studies in mice have shown that COX-2 inhibition decreases implantation after fertilisation.^51^ In humans, prostaglandin synthesis (eg, by COX-2 inhibition) disruption is reported to be associated with repeated IVF (ie, suggesting that reduced prostaglandin synthesis in the human endometrium may lead to poor endometrial receptivity).^52^ Two studies also found a higher risk of pregnancy loss when NSAIDs were used close to conception^47^ or for prolonged treatment.^48^ The American College of Rheumatology has thus conditionally recommended discontinuation of NSAIDs during preconception for patients having difficulty conceiving (and if disease control would not be compromised).^53^

Moreover, Brouwer *et al*^11^ examined fertility in 245 women with RA and found an association between NSAID use during the preconception period and longer TTP. Nonetheless, this association has never been demonstrated in women with SpA. Several articles also show a transient and reversible effect of NSAID use on fertility, with recovery of normal ovulation on cessation of treatment, which makes cessation a valuable modifiable factor.^54^,^56^ Unfortunately, in our study, the small number (n=3) of patients exposed to Cox-2 inhibitors precluded us from performing an analysis stratified by the NSAID class.

The use of biologicals in the preconceptional period was not associated with an increased TTP in our cohort, consistent with the (scarce) data in the literature about their impact on fertility. A retrospective study has even shown that in women with RA desiring to conceive, treatment with biologicals might be associated with a shortening of the TTP.^57^ We also found that disease activity did not have a deleterious impact on TTP in this cohort. Disease activity was generally well controlled during the preconception period in this cohort. Nonetheless, we had only one BASDAI measurement in the preconceptional period for most patients (at the inclusion visit), which hampered our ability to conduct analyses that considered the variation in disease activity over time in patients.

Finally, almost 19% of our patients were current smokers at the time of inclusion, which is in line with French smoking prevalence in the general population of 22% in women.^58^

Our study has several limitations. The statistical power was limited by the relatively small patient sample. Moreover, we could not perform subgroup analyses for the different SpA phenotypes because of the number of participants. The importance of missing data, especially concerning disease activity, may also have hampered the power of our analyses. Nevertheless, we addressed this limitation by using multiple imputations. Some data were not available in the database, such as quality of life and frequency of sexual intercourse, which may affect the primary outcome. Despite the national and multicentre aspect of the cohort, patients were all recruited from tertiary rheumatology centres, which may result in a selection bias in favour of potentially more severe forms of the disease. Nevertheless, although the rheumatology follow-up took place in the tertiary centres, gynaecological and/or obstetric preconceptional and pregnancy care did not do so systematically. This information is not collected in the current version of the clinical research form. Finally, there was insufficient data on the frequency and dose of NSAIDs used in the cohort to refine the results according to these parameters.

Our study also has several strengths worth mentioning. The multicentre national design allowed a more representative cohort. Prospective patient follow-up and data collection limit the biases of retrospective studies and allow us to analyse the effect of SpA treatment exposure and disease activity on fertility. This is particularly important as the literature is almost devoid of data about fertility, and most of it comprises retrospective studies at high risk of bias. The addition of a random centre effect in the statistical models allowed this factor to be considered in the analyses. We also performed a sensitivity analysis to reassess TTP in the overall population. It showed that the TTP and subfertility rate in the preconception population may indeed be overestimated by the selection bias of these patients, as those who became pregnant easily were more likely to be included after pregnancy onset.

Our study shows that women with SpA appear to have a longer TTP and a higher subfertility rate, with the longer TTP associated with maternal age and preconceptional NSAID use. More research is needed to confirm these findings and understand the mechanisms behind this association, as well as to study the role of disease activity in greater depth. TFN-inhibitors, given the increasing evidence of their fertility benefits,^59^ the minimal safety concerns with their use during pregnancy,^60^ and their NSAID-sparing effect,^61 62^ represent an effective and safe treatment alternative for patients receiving continuous NSAIDs who are trying to conceive.

Our findings underline the importance of preconceptional counselling in all patients who desire pregnancy and the need to reduce or cease NSAID intake in SpA patients struggling to conceive.

## supplementary material

10.1136/rmdopen-2024-004745 online supplemental table 1

## Data Availability

Data are available on reasonable request. No data are available.

## References

[1] Carmona L, Cross M, Williams B (2010). Rheumatoid arthritis. Best Pract Res Clin Rheumatol.

[2] Stolwijk C, Boonen A, van Tubergen A (2012). Epidemiology of spondyloarthritis. Rheum Dis Clin North Am.

[3] Reveille JD, Witter JP, Weisman MH (2012). Prevalence of axial spondylarthritis in the United States: estimates from a cross-sectional survey. Arthritis Care Res (Hoboken).

[4] Chimenti M-S, Alten R, D’Agostino M-A (2021). Sex-associated and gender-associated differences in the diagnosis and management of axial spondyloarthritis: addressing the unmet needs of female patients. RMD Open.

[5] Sellam J, Morel J, Tournadre A (2021). PRACTICAL MANAGEMENT of patients on anti-TNF therapy: Practical guidelines drawn up by the Club Rhumatismes et Inflammation (CRI). Joint Bone Spine.

[6] Brouwer J, Laven JSE, Hazes JMW (2013). Levels of serum anti-Müllerian hormone, a marker for ovarian reserve, in women with rheumatoid arthritis. Arthritis Care Res (Hoboken).

[7] Şahin A, Karakuş S, Durmaz Y (2015). Evaluation of Ovarian Reserve with Anti-Müllerian Hormone in Familial Mediterranean Fever. Int J Rheumatol.

[8] Velarde-Ochoa M del C, Esquivel-Valerio JA, Vega-Morales D (2015). Anti-müllerian Hormone in Reproductive Age Women With Systemic Lupus Erythematosus. Reumatol Clin (Engl Ed).

[9] Tuten A, Hatipoglu E, Oncul M (2014). Evaluation of ovarian reserve in Hashimoto’s thyroiditis. Gynecol Endocrinol.

[10] Karakus S, Sahin A, Durmaz Y (2017). Evaluation of ovarian reserve using anti-müllerian hormone and antral follicle count in Sjögren’s syndrome: Preliminary study. J Obstet Gynaecol Res.

[11] Brouwer J, Hazes JMW, Laven JSE (2015). Fertility in women with rheumatoid arthritis: influence of disease activity and medication. Ann Rheum Dis.

[12] Pons M, Dougados M, Costedoat-Chalumeau N (2021). Pregnancy rates and outcomes in early axial spondyloarthritis: An analysis of the DESIR cohort. Joint Bone Spine.

[13] Hamroun S, Hamroun A, Bigna J-J (2022). Fertility and pregnancy outcomes in women with spondyloarthritis: a systematic review and meta-analysis. Rheumatology (Oxford).

[14] Eudy AM, McDaniel G, Clowse ME (2020). Pregnancy outcomes, fertility, and family planning in women with psoriatic arthritis. Obstet Med.

[15] Østensen M (2017). Sexual and reproductive health in rheumatic disease. Nat Rev Rheumatol.

[16] Jesam C, Salvatierra AM, Schwartz JL (2014). Effect of oral administration of a continuous 18 day regimen of meloxicam on ovulation: experience of a randomized controlled trial. Contraception.

[17] Jesam C, Salvatierra AM, Schwartz JL (2010). Suppression of follicular rupture with meloxicam, a cyclooxygenase-2 inhibitor: potential for emergency contraception. Hum Reprod.

[18] Wanders A, Heijde D van der, Landewé R (2005). Nonsteroidal antiinflammatory drugs reduce radiographic progression in patients with ankylosing spondylitis: a randomized clinical trial. Arthritis Rheum.

[19] Adams K, Bombardier C, van der Heijde D (2012). Safety and efficacy of on-demand versus continuous use of nonsteroidal antiinflammatory drugs in patients with inflammatory arthritis: a systematic literature review. J Rheumatol Suppl.

[20] Dougados M, Braun J, Szanto S (2012). Nonsteroidal antiinflammatory drug intake according to the Assessment of SpondyloArthritis International Society Score in clinical trials evaluating tumor necrosis factor blockers: example of etanercept in advanced ankylosing spondylitis. Arthritis Care Res (Hoboken).

[21] Ursin K, Lydersen S, Skomsvoll JF (2021). Factors Associated With Time to Pregnancy in Women With Axial Spondyloarthritis: A Registry-Based Multicenter Study. Arthritis Care Res (Hoboken).

[22] Murarasu A, Guettrot-Imbert G, Le Guern V (2022). Characterisation of a high-risk profile for maternal thrombotic and severe haemorrhagic complications in pregnant women with antiphospholipid syndrome in France (GR2): a multicentre, prospective, observational study. Lancet Rheumatol.

[23] Larosa M, Le Guern V, Guettrot-Imbert G (2022). Evaluation of lupus anticoagulant, damage, and remission as predictors of pregnancy complications in systemic lupus erythematosus: the French GR2 study. Rheumatology (Oxford).

[24] de Frémont GM, Costedoat-Chalumeau N, Lazaro E (2023). Pregnancy outcomes in women with primary Sjögren’s syndrome: an analysis of data from the multicentre, prospective, GR2 study. Lancet Rheumatol.

[25] Meissner Y, Strangfeld A, Costedoat-Chalumeau N (2019). European Network of Pregnancy Registers in Rheumatology (EuNeP)-an overview of procedures and data collection. Arthritis Res Ther.

[26] Meissner Y, Fischer-Betz R, Andreoli L (2021). EULAR recommendations for a core data set for pregnancy registries in rheumatology. Ann Rheum Dis.

[27] Rudwaleit M, Landewé R, van der Heijde D (2009). The development of Assessment of SpondyloArthritis international Society classification criteria for axial spondyloarthritis (part I): classification of paper patients by expert opinion including uncertainty appraisal. Ann Rheum Dis.

[28] Cox CM, Thoma ME, Tchangalova N (2022). Infertility prevalence and the methods of estimation from 1990 to 2021: a systematic review and meta-analysis. *Hum Reprod Open*.

[29] Sieper J, Rudwaleit M, Baraliakos X (2009). The Assessment of SpondyloArthritis international Society (ASAS) handbook: a guide to assess spondyloarthritis. Ann Rheum Dis.

[30] Lukas C, Landewé R, Sieper J (2009). Development of an ASAS-endorsed disease activity score (ASDAS) in patients with ankylosing spondylitis. Ann Rheum Dis.

[31] Liu L, Wolfe RA, Huang X (2004). Shared frailty models for recurrent events and a terminal event. Biometrics.

[32] Juul S, Karmaus W, Olsen J (1999). Regional differences in waiting time to pregnancy: pregnancy-based surveys from Denmark, France, Germany, Italy and Sweden. The European Infertility and Subfecundity Study Group. *Hum Reprod*.

[33] Boivin J, Bunting L, Collins JA (2007). International estimates of infertility prevalence and treatment-seeking: potential need and demand for infertility medical care. Hum Reprod.

[34] Slama R, Hansen OKH, Ducot B (2012). Estimation of the frequency of involuntary infertility on a nation-wide basis. Hum Reprod.

[35] Streuli I, de Mouzon J, Paccolat C (2014). AMH concentration is not related to effective time to pregnancy in women who conceive naturally. Reprod Biomed Online.

[36] Hagen CP, Vestergaard S, Juul A (2012). Low concentration of circulating antimüllerian hormone is not predictive of reduced fecundability in young healthy women: a prospective cohort study. Fertil Steril.

[37] Broekmans FJ, Kwee J, Hendriks DJ (2006). A systematic review of tests predicting ovarian reserve and IVF outcome. Hum Reprod Update.

[38] Nelson SM, Yates RW, Lyall H (2009). Anti-Müllerian hormone-based approach to controlled ovarian stimulation for assisted conception. Hum Reprod.

[39] Wu C-H, Chen Y-C, Wu H-H (2009). Serum anti-Müllerian hormone predicts ovarian response and cycle outcome in IVF patients. J Assist Reprod Genet.

[40] Fraisse T, Ibecheole V, Streuli I (2008). Undetectable serum anti-Müllerian hormone levels and occurrence of ongoing pregnancy. Fertil Steril.

[41] Massé V, Ferrari P, Boucoiran I (2011). Normal serum concentrations of anti-Mullerian hormone in a population of fertile women in their first trimester of pregnancy. Hum Reprod.

[42] Yalçın Bahat P, Kadiroğulları P, Topbas Selcuki NF (2021). Ovarian reserve in patients with ankylosing spondylitis. Arch Gynecol Obstet.

[43] Jukic AMZ, McConnaughey DR, Weinberg CR (2016). Long-term Recall of Time to Pregnancy. Epidemiology.

[44] Gurunath S, Pandian Z, Anderson RA (2011). Defining infertility--a systematic review of prevalence studies. Hum Reprod Update.

[45] Mumford SL, Schisterman EF, Cole SR (2015). Time at risk and intention-to-treat analyses: parallels and implications for inference. Epidemiology.

[46] Sammaritano LR, Bermas BL (2018). Management of pregnancy and lactation. Best Pract Res Clin Rheumatol.

[47] Li D-K, Liu L, Odouli R (2003). Exposure to non-steroidal anti-inflammatory drugs during pregnancy and risk of miscarriage: population based cohort study. BMJ.

[48] Nielsen GL, Sørensen HT, Larsen H (2001). Risk of adverse birth outcome and miscarriage in pregnant users of non-steroidal anti-inflammatory drugs: population based observational study and case-control study. BMJ.

[49] Micu MC, Micu R, Ostensen M (2011). Luteinized unruptured follicle syndrome increased by inactive disease and selective cyclooxygenase 2 inhibitors in women with inflammatory arthropathies. Arthritis Care Res (Hoboken).

[50] Pall M, Fridén BE, Brännström M (2001). Induction of delayed follicular rupture in the human by the selective COX-2 inhibitor rofecoxib: a randomized double-blind study. Hum Reprod.

[51] Agrawal SS, Alvin Jose M (2009). Anti-implantation activity of H2 receptor blockers and meloxicam, a COX-inhibitor, in albino Wistar rats. Eur J Contracept Reprod Health Care.

[52] Achache H, Tsafrir A, Prus D (2010). Defective endometrial prostaglandin synthesis identified in patients with repeated implantation failure undergoing in vitro fertilization. Fertil Steril.

[53] Sammaritano LR, Bermas BL, Chakravarty EE (2020). 2020 American College of Rheumatology Guideline for the Management of Reproductive Health in Rheumatic and Musculoskeletal Diseases. Arthritis Care Res (Hoboken).

[54] Smith G, Roberts R, Hall C (1996). Reversible ovulatory failure associated with the development of luteinized unruptured follicles in women with inflammatory arthritis taking non-steroidal anti-inflammatory drugs. Br J Rheumatol.

[55] Akil M, Amos RS, Stewart P (1996). Infertility may sometimes be associated with NSAID consumption. Br J Rheumatol.

[56] Mendonça LL, Khamashta MA, Nelson-Piercy C (2000). Non-steroidal anti-inflammatory drugs as a possible cause for reversible infertility. Rheumatology (Oxford).

[57] Shimada H, Kameda T, Kanenishi K (2019). Effect of biologic disease-modifying anti-rheumatic drugs for patients with rheumatoid arthritis who hope to become mothers. Clin Rheumatol.

[58] Article - bulletin épidémiologique hebdomadaire. http://beh.santepubliquefrance.fr/beh/2023/9-10/2023_9-10_1.html.

[59] Winger EE, Reed JL, Ashoush S (2009). Treatment with adalimumab (Humira) and intravenous immunoglobulin improves pregnancy rates in women undergoing IVF. Am J Reprod Immunol.

[60] Giles I, Thorne I, Schmidt NS (2024). The time of equipoise on the use of biological DMARDs in for inflammatory arthritis during pregnancy is finally over: a reappraisal of evidence to optimise pregnancy management. Lancet Rheumatol.

[61] Dougados M, Wood E, Combe B (2014). Evaluation of the nonsteroidal anti-inflammatory drug-sparing effect of etanercept in axial spondyloarthritis: results of the multicenter, randomized, double-blind, placebo-controlled SPARSE study. Arthritis Res Ther.

[62] Moltó A, Granger B, Wendling D (2015). Brief Report: Nonsteroidal Antiinflammatory Drug-Sparing Effect of Tumor Necrosis Factor Inhibitors in Early Axial Spondyloarthritis: Results From the DESIR Cohort. *Arthritis Rheumatol*.

