# Supplementary material for: NSAID exposure delays time-to-pregnancy in patients with spondyloarthritis: an analysis of the GR2 prospective cohort
Source: RMD Open. 2024 Nov 29;10(4):e004745. doi: 10.1136/rmdopen-2024-004745 (PMC11624830; doi:10.1136/rmdopen-2024-004745)
Supplement: online supplemental table 1 [file rmdopen-10-4-s001.pdf]

Supplementary Table 1. Description of NSAIDs used preconceptionally.

	**Patients (N = 23)**
**Acetic acids**, n (%)	
Diclofenac, n (%)	2 (8.7%)
Aceclofenac, n (%)	1 (4.3%)
Indomethacin, n (%)	8 (34.8%)
**Propionic acids**, n (%)	
Naproxen, n (%)	3 (13.0%)
Ibuprofen, n (%)	1 (4.3%)
Ketoprofen, n (%)	2 (8.7%)
**Nabumetone**, n (%)	1 (4.3%)
**Phenylbutazone**, n (%)	2 (8.7%)
**Selective Cox-2 inhibitors**, n (%)	
Celecoxib, n (%)	3 (13.0%)

Cox-2: cyclooxygenase 2.
